# Clonal Hematopoiesis and Type 2 Diabetes: A Narrative Review

**DOI:** 10.1111/1753-0407.70251

**Published:** 2026-06-29

**Authors:** Jian‐Jun Liu, Sylvia Liu, Resham L. Gurung, Su Chi Lim, Rajkumar Dorajoo

**Affiliations:** ^1^ Clinical Research Unit Khoo Teck Puat Hospital Singapore; ^2^ Duke‐NUS Graduate Medical School Singapore; ^3^ Saw Swee Hock School of Public Health National University of Singapore Singapore; ^4^ Lee Kong Chian School of Medicine Nanyang Technological University Singapore; ^5^ Genome Institute of Singapore (GIS), Agency for Science, Technology and Research (A*STAR) Singapore; ^6^ Department of Paediatrics, Yong Loo Lin School of Medicine National University of Singapore Singapore

**Keywords:** clonal hematopoiesis, diabetic complications, inflammation, Type 2 diabetes

## Abstract

Clonal hematopoiesis (CH) refers to an expanded clonal hematopoietic cell population due to acquired mutations conferring a selective growth advantage. Once considered a premalignant hematological state, CH has now been causally associated with cardiovascular diseases (CVDs) and several aging‐related clinical conditions. Emerging evidence suggests that CH is at the intersection of aging and metabolic disorders. CH carriers exhibit a higher risk of Type 2 diabetes, potentially through the obesity–inflammation–insulin resistance axis. Moreover, CH confers a higher risk of CVD and is associated with a poorer prognosis in patients with diabetes compared to noncarriers. In addition, CH has been associated with the development of microvascular complications, including retinopathy and kidney disease in patients with diabetes, although inconsistent findings exist. Preclinical studies found that antidiabetic medication metformin might slow down clonal expansion of CH, while clinical data suggest that anti‐inflammatory medications confer better clinical outcomes for certain CH carriers, although their relevance for patients with diabetes remains to be elucidated. In this narrative review, we also briefly discuss the barriers to clinical adoption of CH detection and highlight the knowledge gaps that should be addressed in future work.

## Highlights

Clonal hematopoiesis (CH) is emerging as a potential novel risk factor that drives the onset of Type 2 diabetes. It may also play a key role in the pathogenesis and progression of vascular complications by promoting local and systemic inflammation. Screening for CH and monitoring the clonal expansion may potentially improve risk stratification and guide precision treatment in patients with Type 2 diabetes.

## Graphical abstract

Clonal hematopoiesis (CH), driven by obesity‐ and aging‐related inflammation, may be causally associated with an increased risk of Type 2 diabetes through the inflammation–insulin resistance pathway. By exacerbating systemic inflammation, CH may also contribute to the development of macrovascular and microvascular complications in patients with Type 2 diabetes.

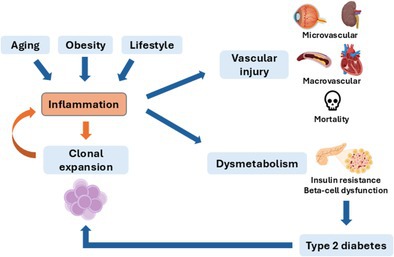

## 1 Clonal Hematopoiesis

Clonal hematopoiesis (CH) refers to the clonal expansion of hematopoietic stem/progenitor cells (HSPCs) due to a de novo mutation that confers a selective growth advantage. The CH driver genes are commonly involved in epigenetic and transcriptional regulation, mRNA splicing, or DNA damage repair [1, 2]. Although a large number of candidate driver genes have been reported, approximately 90% of the mutations occur in one of the following eight genes: *DNMT3A*, *TET2*, *ASXL1*, *JAK2*, *SF3B1*, *SRSF2*, *PPM1D*, and *TP53* [2, 3]. CH is often called clonal hematopoiesis of indeterminate potential (CHIP) when the variant allele frequency (VAF) of the driver mutation exceeds 2% in peripheral leukocytes [4, 5].

CH driver mutations may be detected by whole‐genome sequencing, whole‐exome sequencing (WES), or targeted gene panel sequencing. WES is most frequently applied in epidemiological studies, whereas targeted deep sequencing is required for reliable detection of clones with VAF below 2% [3]. Improved error‐corrected or duplex sequencing approaches may reduce artifacts and improve discrimination of somatic mutations from germline variation.

CH may also arise in the absence of detectable driver gene mutations, especially in elderly people [6, 7]. This may result from neutral drift acting on a small aging population of active HSPCs or that CH is driven by variation in clonally inherited epigenetic states that affect the self‐renewal and proliferative capacities of HSPCs. It is also possible that CH is driven by heterogeneous genetic mutations, where each individual driver mutation occurs at a very low frequency in the CH population [7]. To date, nearly all the studies on CH and cardio‐metabolic diseases focus on CH with known driver mutations.

CH has been considered a hallmark of aging because age is the strongest determinant of CH prevalence [8]. Male sex, smoking status, poor diet quality, history of atherosclerotic cardiovascular disease (CVD), cancer treatments, and germline genetic predispositions are also associated with a high prevalence of CH [8, 9, 10, 11]. Therefore, the prevalence of CH highly depends on the characteristics of the study population, especially age and comorbidities, sequencing depth, and the VAF threshold for the definition of CH. For example, CHIP (VAF > 2%) may be identified in less than 1% of people younger than 40 years old, while it may be detected in up to 20% of individuals 70 years and older [4, 12]. Using sensitive sequencing methods with VAF > 0.5% as the threshold, Assmus et al. found that CH was prevalent in 56% of participants with chronic ischemic heart failure [13].

CH was once considered a premalignant hematological state. A landmark study in 2014 found that, while the presence of CH conferred a 30%–40% higher risk for all‐cause mortality, it was the increased cardiovascular death rather than hematologic malignancy that accounted for the excessive mortality [2]. Subsequently, CH has been associated with a wide range of cardiovascular disorders, including coronary artery disease, heart failure, atrial fibrillation, stroke, and CVD mortality, independent of conventional risk factors [2, 4, 5, 12, 14]. Besides CVD, CH has also been linked with an increased risk for infectious disease, autoimmune conditions, solid organ tumors, acute and chronic kidney diseases, and other aging‐related disorders [15, 16, 17].

It is generally believed that CH promotes the pathogenesis and progression of various diseases mainly through the development of a pro‐inflammatory state [4, 17, 18, 19]. Notably, CH derived from mutations of different driver genes may elevate the tone of inflammation via distinct pathways. For example, *TET2* mutations increase the tone of inflammation by the NLR family pyrin domain‐containing 3 (NLRP3) inflammasome pathway with the release of interleukin‐1β (IL‐1β), IL‐18, and the upregulation of IL‐6 as effectors [20]. Beyond the NLRP3–IL‐1β axis, *DNMT3A* mutations may also activate inflammation by upregulating Type I interferon responses, potentially through mechanisms such as mitochondrial DNA sensing or by promoting monocyte–T cell interaction via increased expression of T‐cell‐stimulating immunoglobulin superfamily members, including CD300LB, CD83, SIGLEC12, and CD2 ligand [21, 22]. On the other hand, CH arising from ASXL1 mutation may activate the NF‐κB pathway through loss of inhibition of IRAK1–TAK1 [23], while CH attributable to *JAK2* mutation may drive inflammation via activation of the AIM2 inflammasome pathway [24, 25].

## 2 CH, Obesity, and Risk of Type 2 Diabetes

The prevalence of obesity is rising rapidly in both developed and developing countries due to changes in demographics, environment, and lifestyle. Several lines of evidence support that CH is associated with obesity. In the UK Biobank cohort, participants with prevalent CH have an increased waist‐to‐hip ratio [26]. In the Women's Health Initiative study, obese participants had higher odds of being detected with CH compared to those with a normal body mass index. The relationship between obesity and CH may be bidirectional. Obesity, especially central obesity, promotes systemic inflammation, which favors the expansion of mutant HSPCs, especially for cells with *TET2*, *DNMT3A*, *ASXL1*, and *JAK2* mutations [26]. Conversely, clonal expansion of CH may promote obesity and exacerbate inflammation, as suggested by a recent study in the UK Biobank cohort and the All of Us cohort. The meta‐analysis of data from these two cohorts showed that CH driven by a DNMT3A mutation was associated with a 14% higher risk of incident obesity in the lean people (HR: 1.14 [1.01–1.27]) [27].

It is well‐established that obesity and the associated systemic inflammation are closely associated with the risk of diabetes, mainly Type 2 diabetes. As a corollary, several clinical studies have associated CH and the risk of Type 2 diabetes. An early study reported that clonal expansion of chromosomal alterations in white blood cells, with some of them affecting CH‐driver genes, was associated with a higher prevalence of Type 2 diabetes [28]. This was followed by another cross‐sectional study showing 30% higher odds of prevalent Type 2 diabetes in CH carriers compared to noncarriers [2]. Consistent with this, the prospective National Heart, Lung, and Blood Institute Trans‐Omics for Precision Medicine (TOPMed) study (*n* = 17 637) reported that CH carriers had a 23% higher risk of incident Type 2 diabetes than noncarriers. Findings in this large‐scale study also highlighted that “not all CHs are equal.” The associations with incident Type 2 diabetes were statistically significant in CH carriers with *TET2* (HR: 1.48 [1.05–2.08]) and *ASXL1* (HR: 1.76 [1.03–2.99]) mutations, while the association between *DNMT3A* mutation and incident Type 2 diabetes was not statistically significant (HR: 1.15 [0.93–1.43]) [29]. Notably, Zhao et al. reported that the presence of *TET2*‐driven CH was not causally associated with an accelerated onset of Type 2 diabetes in their two‐sample Mendelian randomization analysis. Instead, prevalent Type 2 diabetes increased the propensity for the development of *TET2* CH [30]. Hence, more clinical studies are needed to infer the causal relationship between CH and Type 2 diabetes.

**FIGURE 1:**
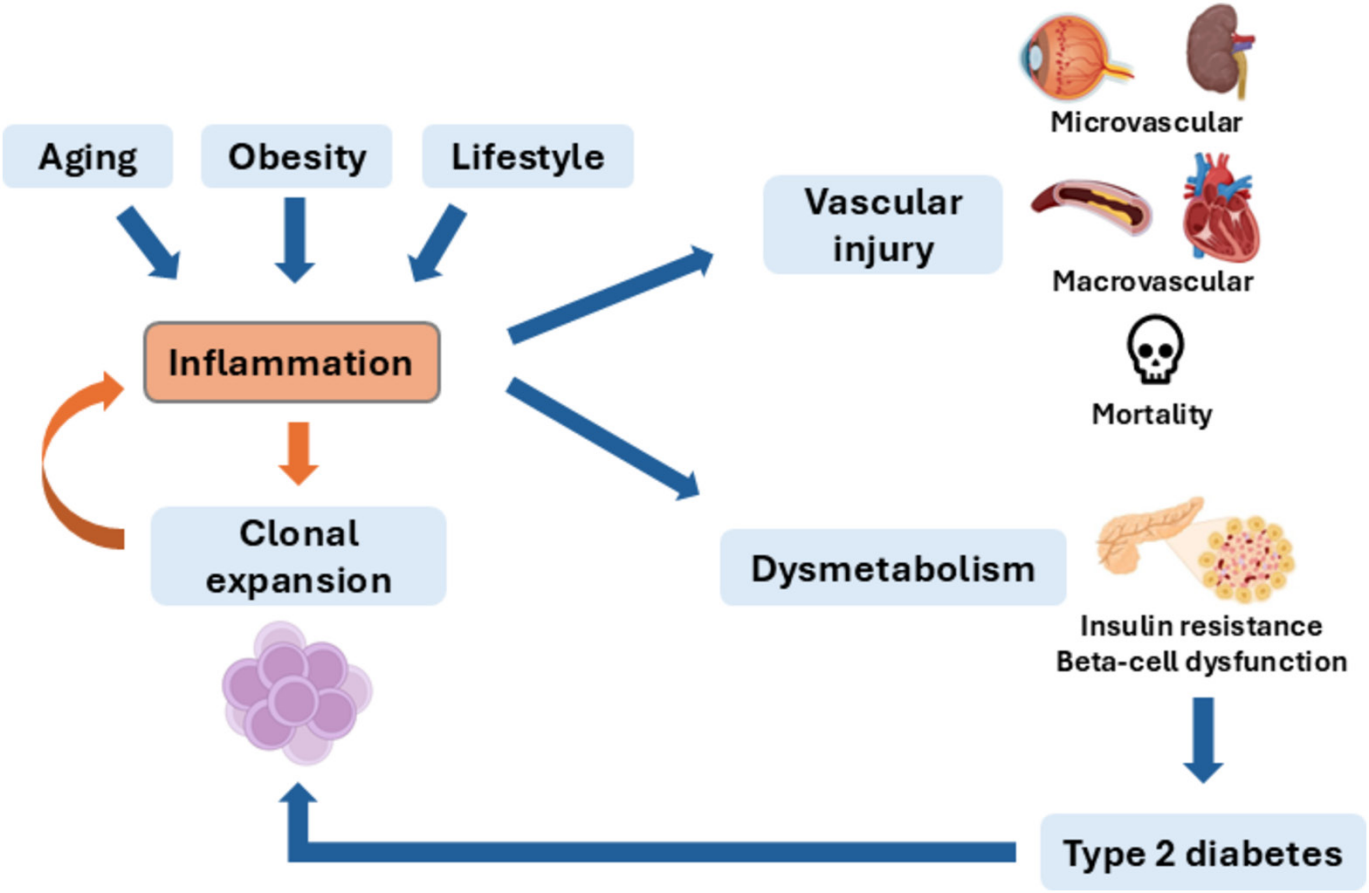
Schematic diagram showing the potential interrelationship between clonal hematopoiesis and the development of Type 2 diabetes and vascular complications.

Mechanistically, the onset of Type 2 diabetes results from an imbalance between insulin secretion and insulin sensitivity. An elegant preclinical study found that mice with *TET2*‐mutant blood cells developed obesity‐related insulin resistance accompanied by increased expression of pro‐inflammatory cytokine IL‐1β in adipose tissue. Pharmacological inhibition of IL‐1β production alleviated the obesity‐related insulin resistance. These findings implicate *TET2* loss‐of‐function as a critical mediator in the obesity–inflammation–insulin resistance axis [31]. Similarly, a recent preclinical study reported that mice carrying *DNMT3A*‐mutant hematopoietic cells exhibited faster body weight gain (1.7‐fold) and a moderately higher food intake compared with wild‐type controls even on a normal chow diet. When these mice were maintained on a high‐fat diet, they demonstrated an elevated fasting blood glucose, a higher level of plasma insulin, and impaired glucose tolerance, accompanied by increased abundance of inflammatory monocytes in spleens and peripheral blood. These data suggest that CH driven by *DNMT3A* mutation may directly contribute to metabolic dysfunction and increase the risk of Type 2 diabetes [27], although the relevance of these findings in humans remains to be elucidated. It is reasonable to hypothesize that CH mediates the association between aging, obesity, and increased risk of Type 2 diabetes by driving systemic or local inflammation, ultimately leading to insulin resistance, β‐cell dysfunction, and development of Type 2 diabetes [31] (Figure 1). Future preclinical and clinical studies are warranted to test this hypothesis.

## 3 CH and Diabetic Complications

The association between prevalent CH and risk of CVD is well‐established in the general population and in individuals with high cardiovascular risk [2, 4, 5, 12, 14]. Patients with Type 2 diabetes experience a twofold to fourfold increased risk of CVD compared to nondiabetic counterparts, even after accounting for traditional clinical risk factors [32]. Emerging evidence suggests that CH may play a role in the pathogenesis of CVD in patients with diabetes. In the UK Biobank, participants free of prevalent CVD, individuals with diabetes, and those with any CH experienced an 18% higher risk of incident coronary artery disease and a 73% higher risk of incident heart failure during a median of 13 years of follow‐up. Further analysis showed that the associations between *SF3B1* and *TET2*, CH, and CVD appeared more pronounced (HR: 2.50 [1.25–5.01] and 1.36 [1.07–1.77], respectively) [33]. The association between CH and increased risk of heart failure found in this study is of particular interest. Most patients with heart failure are diagnosed late, requiring a paradigm shift toward prevention, risk stratification, and early diagnosis [34]. Given the close relationship between CH and heart failure, identifying and targeting CH may be a promising preventive strategy in both nondiabetic and diabetic populations. In another cohort study in patients with a history of myocardial infarction, the prevalence of CH was 1.43‐fold higher in patients with Type 2 diabetes compared to nondiabetic counterparts. Moreover, the mortality rate was doubled in patients with Type 2 diabetes and CH compared to their counterparts with diabetes who were non‐CH carriers, suggesting that CH might confer a poorer prognosis in this high‐risk population [30]. Although it is biologically plausible that CH may interact with Type 2 diabetes to amplify the risk of mortality by increasing inflammatory responses and immune dysregulation, the authors did not formally test the statistical significance of an interaction term in that study.

On the other hand, CH may also play a role in microvascular complications in patients with Type 2 diabetes. In UK Biobank participants with Type 2 diabetes (*n* = 20 712), the presence of any CH was associated with a 1.34 (1.13–1.57)‐fold increased risk for the development of retinopathy and a 1.26 (1.10–1.45)‐fold higher risk for incident kidney disease independent of clinical risk factors. The risk of microvascular complications is especially high for CHs driven by *DNMT3A*, *TET2*, and *NF1* mutations [35]. Another nested case–control study (*n* = 294) also examined the association between CH and risk of adverse kidney outcomes in patients with Type 2 diabetes. However, the authors did not observe a significant association between prevalent CH and kidney outcome defined as a composite of incident kidney disease or progression [36]. It was postulated that the differences in event definition, sequencing depth, CH calling algorithms, and the relatively small sample size might have biased the analysis toward a nonsignificant association [37].

Chronic inflammation is a common pathway leading to vascular diseases in both diabetic and nondiabetic populations [38, 39]. Chronic inflammation accelerates vascular pathology, including endothelial injury, lipid accumulation, plaque destabilization, and thrombosis [40]. Given the close relationship between CH and chronic inflammation as discussed above [4, 18, 19], it is reasonable to hypothesize that the association between CH and vascular complications in patients with diabetes is at least partly mediated by high levels of systemic or local inflammation (Figure 1). Notably, preclinical studies found that CH might also drive cardiac fibrosis through mechanisms beyond inflammation [41, 42], suggesting that the pathophysiologic linkage between CH and diabetic complications can be multifaceted.

The major clinical studies on the associations between CH, Type 2 diabetes, and its complications are summarized in Table 1.

**TABLE 1:** Major clinical studies on the associations between CH, Type 2 diabetes development, and vascular complications.

Authors [reference]	Design and study population	Study outcomes	Main driver mutations	Main findings
Type 2 diabetes development				
Jaiswal et al. (2014) [2]	Cross‐sectional analysis in 17 182 persons without hematologic disorders	Age‐related health outcomes	Absence vs. any CH	1.3‐fold increased odds of prevalent Type 2 diabetes in CH carriers compared to noncarriers
Tobias et al. (2023) [29]	Prospective cohort study; 17 637 participants from TOPMed cohorts, mean follow‐up 9.8 years	Incident Type 2 diabetes	*DNMT3A*, *TET2*, *ASXL1*, *JAK2*, *TP53*	23% higher risk of Type 2 diabetes (HR: 1.23; CI: 1.04–1.45) in carriers of any CH vs. noncarriers. Driver mutation‐specific risks: *TET2* (HR: 1.48 [1.05–2.28]), *ASXL1* (HR: 1.76 [1.03–2.99]), nonsignificant for *DNMT3A*, *TP53*, and *JAK2* carriers
Zhao et al. (2025) [30]	Cross‐sectional analysis in 1430 patients with prior myocardial infarction	CH prevalence and outcomes in patients with versus without Type 2 diabetes	42 CH mutations, with a focus on *TET2*, *ASXL1*	Higher CH prevalence in patients with versus without Type 2 diabetes (10.6% vs. 7.4%). Mendelian randomization suggested that Type 2 diabetes increased CH propensity, but CH did not accelerate Type 2 diabetes onset
Diabetic complications				
Sun et al. (2025) [33]	Prospective cohort study; 22 239 adults with diabetes, median follow‐up 13.2 years	CVD (coronary artery disease, heart failure, stroke)	Any CH and top 10 commonly mutated driver genes	Any CH and incident CVD (HR: 1.21 [1.08–1.36]); of specific driver mutations: *SF3B1* (HR: 2.50 [1.25–5.01]) and *TET2* (HR: 1.36 [1.07–1.77]) were significantly associated with CVD
Zhao et al. (2025) [30]	Prospective cohort; 1430 patients with prior myocardial infarction	All‐cause and cardiac mortality	Any CH; *TET2*, *ASXL1*	2.03‐fold higher all‐cause mortality in Type 2 diabetes with any CH (HR: 2.03 [1.07–3.84]) vs. non‐CH. *ASXL1* CH was significantly associated with cardiac death (HR: 4.51 [1.30–15.6]) in patients with Type 2 diabetes
Wei et al. (2025) [35]	Prospective cohort; 20 712 individuals with Type 2 diabetes	Diabetic microvascular complications (DMCs, retinopathy, kidney disease, neuropathy)	Any CH, common specific driver mutations	Any CH was associated with 1.23 [1.10–1.38] folds high risk of DMCs, specifically diabetic retinopathy and diabetic kidney disease, but not diabetic neuropathy; *TET2*, *NF1*, and *DNMT3A* were significantly associated with the composite DMCs outcome
Denicolo et al. (2022) [36]	Nested case–control study in diabetic patients; 64 cases matched with 230 controls	Composite of kidney replacement, renal death, eGFR decline, and incident macroalbuminuria	46 CH driver genes	CH prevalence was 29% in patients with kidney events. Total CH was not associated with the composite kidney outcome (HR: 1.06 [0.57–1.96]). No specific CH driver mutations were significantly associated with the kidney outcome either

## 4 CH and Medicinal Treatments in Patients With Diabetes

With an in‐depth understanding of the pathophysiology associated with CH, one may expect to target CH‐associated pathways to improve clinical outcomes. CH primarily drives disease progression through pro‐inflammatory signaling. For example, *TET2* loss‐of‐function activates the NLRP3–IL‐1β axis and potentially promotes diabetic complications [33, 35]. This raises the possibility that *TET2* CH carriers may respond better to anti‐inflammatory therapies targeting IL‐1β [5]. A post hoc analysis of the Canakinumab Anti‐inflammatory Thrombosis Outcomes (CANTOS) trial found that the IL‐1β neutralizing antibody canakinumab reduced major adverse cardiovascular events by 62% in patients with *TET2* CH compared to a 7% reduction in noncarriers [18], suggesting that identification of CH due to specific gene mutation may guide precision medicinal treatment. Ongoing trials with colchicine, IL‐6 antagonists, and NLRP3 inhibitors may further inform mutation‐specific treatment strategies [43, 44]. It should be highlighted that the clinical trials mentioned above, including the CANTOS study, are not designed specifically for the diabetic population. Given that inflammation is a hallmark of Type 2 diabetes and an essential driver for diabetic complications [39], the clinical value of CH detection to guide precision diabetes medicine deserves further investigation. On the other hand, sodium–glucose cotransporter‐2 inhibitors and glucagon‐like peptide‐1 receptor agonists also exhibit anti‐inflammatory properties [38]. It may be worthwhile to evaluate whether these agents have better effects for the prevention of complications in CH‐positive patients with diabetes.

Emerging data suggest that CH itself may potentially be a direct therapeutic target for the prevention and treatment of various diseases, including diabetes. For example, recent preclinical studies have found that metformin decreases the survival advantage of *DNMT3A*‐mutated HSPCs by inhibiting mitochondrial oxidative phosphorylation [45, 46]. These findings provide a rationale for investigating metformin as a preventive intervention against *DNMT3A* mutation‐driven CH in humans [47]. This is partly supported by an observational study in the UK Biobank cohort (*n* = 412 234) showing that individuals taking metformin had a markedly lower prevalence of *DNMT3A*‐R882‐mutant CH after accounting for potential confounders, including glycated hemoglobin and body mass index [46]. Hence, it is worthwhile to study whether metformin prevents Type 2 diabetes or improves blood glucose control in patients with prevalent diabetes by restraining CH expansion driven by the *DNMT3A* mutation. On the other hand, new technologies allow containment of CH clone expansion or direct elimination through chimeric antigen receptor T‐cell therapy (CAR‐T) [48] or bispecific T‐cell engagers [49]. The clinical relevance of these new technologies for CH‐based treatments has not yet been explored in either diabetic or nondiabetic populations.

## 5 Future Directions

Compared to non‐CH carriers, patients with diabetes and CH exhibit increased risk of CVD [33], retinopathy [35], and excess mortality [30], independent of conventional risk factors. Hence, CH detection holds the promise for identifying high‐risk patients and tailoring therapies, particularly those agents targeting specific pathways of inflammation, to improve clinical outcomes [18, 50]. In this context, it is worthwhile to assess whether identification of CH status may improve the accuracy of risk stratification and guide precision treatments in diabetes care.

Heart failure and kidney diseases are common causes of morbidity and mortality in the diabetic population [51, 52]. Although CH has been associated with a higher risk of incident heart failure and poorer prognosis in those with prevalent heart failure in the nondiabetic population [53, 54], the role of CH in heart failure has not been systematically characterized specifically in patients with diabetes. Similarly, CH has been associated with fast kidney function decline and increased risk for progression to end‐stage kidney disease in the general population and CKD population [16, 55, 56]. Clinical data on the association between CH and diabetic kidney disease are scarce and inconclusive [36, 37].

Several technical barriers remain for CH detection and the subsequent clinical adoption. CH clone identification requires next‐generation sequencing. It is not yet widely available in routine diabetes care, and the cost‐effectiveness of CH screening is unknown. Also, differentiating true CH mutations from sequencing artifacts and germline variants is still a considerable bioinformatic challenge [57]. Standardization of variant calling is essential for both epidemiological studies and CH diagnostic assays [58]. Furthermore, there is no consensus on VAF thresholds that should trigger clinical concern, particularly for small clones with VAF < 2% [59, 60]. Nevertheless, pioneering multidisciplinary clinics have been set up in some institutes to manage patients with CH [61]. Further research is needed to establish evidence‐based guidelines and intervention strategies for CH carriers. Well‐designed clinical trials stratified by CH status are required to determine whether mutation‐specific interventions can effectively improve diabetes care.

Finally, most CH studies have been conducted in European ancestry cohorts. The prevalence, mutation spectrum, and clinical impact of CH in other racial and ethnic populations remain insufficiently understood. Expanding CH research to diverse ancestries is essential, not only for global generalizability but also to uncover novel biology that may interact with ancestry‐specific metabolic and environmental risk factors.

## 6 Conclusion

CH is emerging as a potential risk factor for metabolic disorders. Research on the role of CH in diabetes and its complications is still in its infancy. Observational studies suggest that diabetes and the related pathophysiological processes may promote clonal expansion of CH, and vice versa, CH may drive the onset of Type 2 diabetes, exacerbate metabolic dysregulation, and promote vascular and nonvascular complications. Nevertheless, causality between CH and diabetes, as well as its complications, is not yet resolved. Future studies are warranted to determine whether CH can serve as a biomarker for risk stratification and a therapeutic target for intervention in diabetes care.

## Funding

This work was supported by KTPH STAR Grants (25203 and 26103) and Singapore NMRC (MOH‐001327‐02, 001704‐00, and 001688‐00).

## Conflicts of Interest

The authors declare no conflicts of interest.

## Data Availability

Data sharing not applicable to this article as no datasets were generated or analysed during the current study.
